# In silico and experimental assessment of the 16S-ITS-23S operon as a genetic marker for high-resolution bacterial species identification

**DOI:** 10.1099/mgen.0.001793

**Published:** 2026-07-24

**Authors:** Ana C. Yamakawa, Matthew Davies, Hyun S. Gweon

**Affiliations:** 1 School of Biological Sciences, University of Reading, Reading, UK; 2 Killgerm Chemicals Ltd, Ossett, UK

**Keywords:** bacterial marker, Oxford Nanopore Technologies (ONT), rRNA operon, taxonomic resolution

## Abstract

Accurate species-level identification of bacteria is crucial for public health surveillance, but standard methods like 16S rRNA hypervariable region (HVR) sequencing often lack sufficient resolution. This study systematically evaluates the taxonomic resolving power of the full 16S-ITS-23S ribosomal RNA (*rrn*) operon, enabled by high-accuracy long-read technology, compared to the full-length 16S gene and HVRs. A dual-validation framework was employed, combining an *in silico* analysis of a curated reference database with an experimental validation using rat faecal samples. In silico, the *rrn* operon demonstrated unequivocally superior accuracy across the full range of tested thresholds for both nucleotide mismatches and pairwise identity, consistently maintaining the highest proportion of monospecies clusters (i.e. clusters containing a single species). For instance, at a relaxed threshold of 30 mismatches, the operon maintained 96.8% monospecies clusters, compared to 80.5% for the full-length 16S gene and <66% for all HVRs. This theoretical advantage was confirmed experimentally through three parallel sequencing strategies: operon sequencing enabled species-level taxonomic assignment for 75.2% of unique sequences, significantly outperforming full-length 16S (62.1%) and dramatically surpassing the standard V4 HVR approach (18.5%). An internal bias-control analysis confirmed that these differences were due to the superior information content of the operon marker itself. Our findings provide robust evidence that high-accuracy long-read sequencing of the *rrn* operon is a superior method for culture-free bacterial surveillance, offering a new gold standard for high-resolution taxonomic profiling in complex environmental and host-associated samples.

**Impact Statement**

Routine bacterial surveillance often relies on sequencing hypervariable regions from the 16S rRNA gene, but many related species can have identical sequences, limiting surveillance and outbreak investigations. This study shows that sequencing the full *rrn* operon with high-accuracy long reads results in a substantially better species-level identification when compared to the full-length 16S or its regions. Using a dual-validation framework, by first benchmarking using operon sequences from a curated reference database and then confirming findings in rat faecal samples, we demonstrate consistently higher taxonomic resolution across multiple clustering thresholds and a greater proportion of monospecies (single-species) groupings. Experimentally, operon sequencing enabled species-level assignment for 75% of unique sequences, outperforming full-length 16S and far exceeding V4-based profiling. Together, these results provide strong, practical evidence for adopting long-read *rrn* operon sequencing as a new culture-free gold standard for high-resolution bacterial profiling in complex host-associated and environmental samples.

## Data Summary

Sequencing data have been deposited to NCBI BioProject accession number PRJNA1320725. Detailed metadata and accession numbers for the 16S-ITS-23S, 16S and V4 datasets analysed in this study are provided in Table S8.

## Introduction

Accurate species-level identification is the cornerstone of effective public health surveillance, particularly for zoonotic diseases that emerge at the human-animal-environment interface. Under the One Health framework, integrating animal and human health monitoring is the most effective strategy to prevent outbreaks and mitigate economic burdens [1, 2]. However, the success of this strategy is fundamentally dependent on the resolving power of the tools used. Whilst a wide range of techniques exist, from conventional culturing to targeted PCR, they often lack the breadth for comprehensive surveillance of unknown or diverse bacterial taxa [3–10]. The inherent limitations of traditional culturing, particularly the difficulty in capturing unknown or diverse pathogens, have necessitated a widespread shift towards culture-independent sequencing methods for comprehensive health surveillance [1, 5, 8]. However, the success of this strategy is fundamentally dependent on the resolving power of the tools employed, as standard sequencing approaches frequently fail to achieve the reliable species-level identification required to distinguish virulent pathogens from their closely related but benign commensal relatives [4, 11].

For decades, the 16S rRNA gene has been the most widely used genetic marker for bacterial phylogenetics [4, 12]. Its universal presence in bacteria and its structure of conserved and hypervariable regions (HVRs) make it an ideal target. Next-generation sequencing of HVRs, such as the V4 region, enables high-throughput profiling of microbial communities, where established benchmarks for sequencing depth have traditionally allowed for broad characterization of community composition [13, 14]. However, the reliance on these short reads (~150–300 bp) severely limits taxonomic resolution, and whilst effective for characterizing community compositions at the family or genus level, HVR sequencing is generally insufficient for the accurate species-level classification required for definitive pathogen identification [11, 12, 15].

The advent of third-generation sequencing, such as the Oxford Nanopore Technologies (ONT) [16–19] and PacBio platforms [15, 20], has enabled the sequencing of the full-length 16S rRNA gene, providing a significant improvement in taxonomic resolution over short-read HVRs [19]. Whilst historical concerns regarding the high error rates of long-read technologies once limited its application for single-nucleotide-sensitive amplicons [16, 21–25], recent advancements in flow cell chemistry (R10.4.1) and basecalling algorithms have pushed sequencing accuracy to ~99% [26]. This leap in fidelity, comparable to established short-read platforms [18, 27], has renewed interest in even longer targets, specifically the ~4.5 kb 16S-ITS-23S ribosomal RNA (*rrn*) operon.

The *rrn* operon contains not only the 16S gene but also the highly variable internal transcribed spacer (ITS) and the 23S gene. Recent *in silico* studies have suggested that this region provides significantly more phylogenetic information than the 16S gene alone, particularly for complex families such as Lactobacillaceae [28, 29]. These theoretical advantages have been supported by experimental studies using mock communities and niche clinical isolates, where the operon achieved species-level assignment for 98–100% of reads compared to 68–80% for 16S-based methods [29, 30]. Furthermore, the operon has shown promise in specialized applications, from hydroponic farming [31] and dairy fermentation [32] to tracking strain-level engraftment in faecal microbiota transplants [33, 34].

However, the transition towards operon-based identification has exposed significant dependencies on database quality and methodological bias. Identification accuracy is fundamentally constrained by the reference repository used; many current databases are heavily biassed toward biomedical entries, which can lead to a 32–82% reduction in strain-level identification for environmental samples and an overall underestimation of global microbial diversity [35]. Whilst benchmarks using mock communities suggest that platform selection among high-accuracy long-read technologies (e.g. ONT vs PacBio) has a limited impact on species-level profiles, the choice of reference database remains a significant variable in achieving consistent assignments [36]. Specifically, mapping against curated, quality-checked collections such as the GROND database has been shown to yield the most accurate results across diverse sequencing platforms [37].

Despite the theoretical superiority of the operon, practical application in complex samples has revealed potential pitfalls. Targeted amplicon methods have successfully identified pathogens in equine gut and human milk microbiomes, yet they remain vulnerable to underestimating taxa with unlinked rRNA genes or suffering from compositional biases in mock communities [38, 39]. Whilst these studies have established the operon’s utility in specific clinical or niche environments, many relied on cross-platform comparisons such as benchmarking Nanopore operon data against short-read Illumina HVR data. This introduces a significant confounding factor as it remains unclear if the observed performance gains are a result of the genetic information in the operon itself or simply a result of disparate sequencing chemistries and error profiles. Furthermore, although shotgun metagenomics is generally considered the ‘gold standard’ for ground-truth community composition by eliminating PCR biases, its higher costs and computational requirements may make the *rrn* operon a more practical and accessible alternative for large sample batch.

To address these methodological uncertainties, this study provides a comprehensive validation that explicitly decouples genetic information content from sequencing technology bias. Building on previous research, we first performed a domain-wide *in silico* analysis across 30 phyla using a curated GROND database to establish a theoretical baseline for the operon’s universal resolving power. We then provided a robust experimental validation in complex brown rat faecal samples (*Rattus norvegicus*). Crucially, to isolate the effect of the genetic marker from the confounding variables of sequencing platform and chemistry, we bioinformatically extracted 16S and HVR sequences directly from the same high-quality Nanopore operon reads. By holding the sequencing platform and the specific read set constant, this approach effectively isolates the genetic marker as the sole variable. This allows us to definitively attribute the operon’s superior identification accuracy to its greater genetic information content, rather than to differences in sequencing chemistry or error profiles that have confounded previous comparisons.

## Methods

**Fig. 1.**
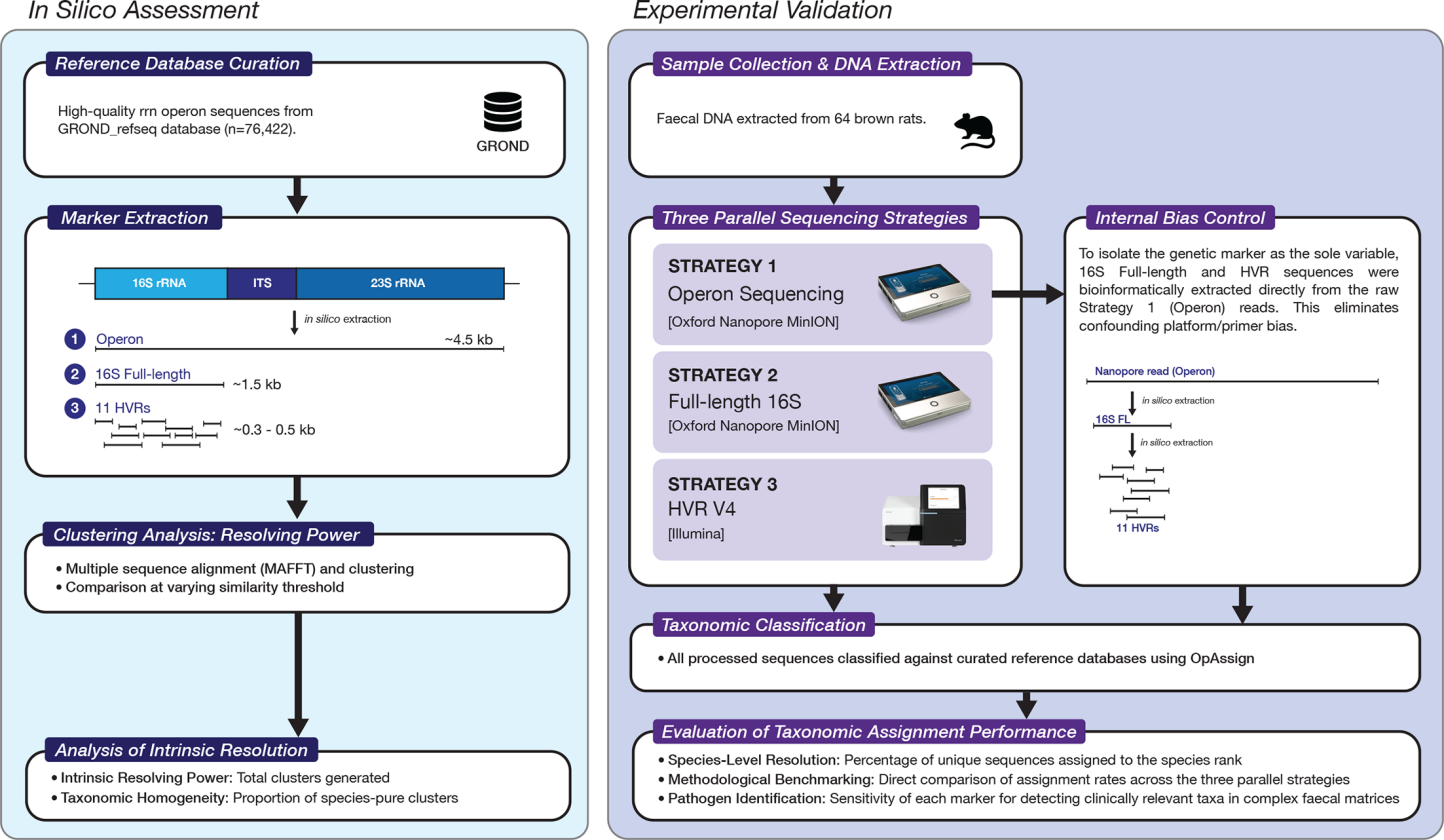
Schematic workflow for the comparative assessment of the *rrn* operon as a genetic marker for bacterial identification. The study framework is divided into two primary analytical domains designed to differentiate theoretical resolving power from practical performance. (Left panel) In Silico Assessment: a curated reference database (GROND_refseq) was used to establish a theoretical baseline. Following bioinformatic marker extraction and normalization (*n*=54, 522 matched taxa), sequences were subjected to clustering analysis across varying similarity thresholds (mismatches and PID) to quantify intrinsic resolving power (total unique clusters) and taxonomic homogeneity (proportion of monospecies clusters). (Right panel) Experimental Validation: real-world performance was evaluated using faecal DNA from 64 brown rats (*R. norvegicus*). Three Parallel Sequencing Strategies were employed: (1) *rrn* operon on Oxford Nanopore (ONT), (2) full-length 16S on ONT and (3) V4 HVR on Illumina. Internal Bias Control: to definitively isolate the genetic marker as the sole variable, 16S and HVR sequences were bioinformatically extracted directly from the raw Strategy 1 (Operon) reads. This decouples information content from platform/primer bias, enabling a direct head-to-head comparison of assignment accuracy using the OpAssign classifier. The final evaluation measures species-level resolution and the sensitivity of each marker for bacterial identification in complex host-associated matrices.

Fig. 1 provides an overview of the analytical framework used to benchmark the *rrn* operon as a genetic marker for bacterial identification. The workflow comprises complementary *in silico* and experimental components, which are described in detail in the following subsections.

### *In silico* evaluation of taxonomic resolving power

#### Database curation and normalization

An *in silico* analysis was conducted to theoretically assess the taxonomic resolving power of the *rrn* operon, the full-length 16S rRNA gene and 11 distinct 16S HVRs. The analysis utilized the GROND_refseq database, a curated collection of *rrn* operon sequences from NCBI RefSeq genomes [37]. To ensure data quality, the database was further curated by removing sequences containing degenerate nucleotides or non-contiguous operons. This process yielded a final database of 76,422 high-quality operon sequences, hereafter referred to as op_GROND_refseq. From this primary extra curated database, corresponding full-length 16S rRNA gene sequences were extracted using Infernal v 1.1.5 [40] and the ext_infernal_16S_id.py script (all custom scripts are available at GitHub: https://github.com/hsgweon/opassign). The extracted sequences were subsequently filtered for length (1,300–1,950 bp) using SeqKit v 2.8.2 [41], creating the 16S_GROND_refseq dataset with 76,382 sequences (99.9% of the op_GROND_refseq). To extract the 11 HVRs (Table S1, available in the online Supplementary Material), HVR-specific blast databases were first constructed. HVR sequences were initially sourced from the Greengenes database [42] and isolated using 11 specific primer pairs with SeqKit v2.8.2. Primer sequences were then removed using Cutadapt v4.9 [43]. These curated HVR sequences were formatted into blast-compatible databases using makeblastdb. The 16S_GROND_refseq sequences were subsequently aligned against these HVR-specific databases using blastn v2.13.0 [44, 45], which retrieved an average of 73,565 corresponding HVR sequences (Table S2). The use of HVR-specific blast databases is a more conservative approach, aiming to avoid specific primer limitation and maximize the retrieval of HVR sequences. To ensure a direct and fair comparison, all datasets (*rrn* operon, full-length 16S rRNA and 11 HVRs) were normalized to retain only the 54,522 taxa for which a sequence was successfully retrieved for every marker, representing 71.34% of the op_GROND_refseq. This normalization was performed using the fastaretrieveseqs.py script. Whilst primer bias is a critical variable in practical applications, this normalization was necessary to isolate the intrinsic genetic resolving power of each region as the sole variable and avoid taxa-related bias in our comparisons. The final dataset consisted of 30 different phyla, 53 classes, 133 orders, 315 families, 1,342 genera and 4,453 species.

#### Analysis of resolving power and taxonomic accuracy

The ability of each genetic marker to resolve distinct bacterial species was evaluated through a two-step clustering analysis. This approach was designed to first quantify the intrinsic sequence diversity captured by each marker and then to assess whether this diversity translated to accurate species-level differentiation. First, to measure the intrinsic resolving power, sequences for each marker were aligned using MAFFT v7.525 with the FFT-NS-2 algorithm [46, 47]. The total number of sequence clusters was then calculated from these alignments at progressively relaxed similarity thresholds, using two metrics: nucleotide mismatches (1 to 30) and pairwise identity (100%–91%; PID). The total number of clusters generated by a marker provides a quantitative measure of its sequence variability at a given mismatch or PID threshold. Since each cluster represents a distinct grouping of similar sequences, a marker that yields a higher total number of clusters demonstrates a greater capacity to resolve closely related sequences that could otherwise be erroneously merged into a single operational taxonomic unit (OTU). Second, to evaluate taxonomic accuracy, the species-level identity of the sequences within each generated cluster was examined. This step was performed to validate that the observed sequence-level resolution was biologically meaningful. A cluster was defined as ‘monospecies’ if all its constituent sequences belonged to a single species. The number of such monospecies clusters was calculated for each marker across all similarity thresholds. This analysis is critical because a high total cluster count is only valuable for classification if the clusters accurately represent distinct species. This second metric therefore measures a marker’s ability to correctly group related sequences without erroneously combining different species, a key requirement for a reliable genetic marker. Both analyses were conducted using the run_taxonomic_resolution_pipeline_improved.py script on the full normalized dataset and on subsets for three well-known pathogenic families: Enterobacteriaceae, Listeriaceae and Campylobacteraceae. Results were visualized using the ggplot2 package v3.4.3 in R.

### Analysis of rat faecal samples

#### Sample collection and DNA extraction

A total of 64 brown rat (*R. norvegicus*) faecal samples were collected by volunteer pest controllers from urban, rural and suburban environments across the UK between January 2023 and May 2023. Samples were stored in 15 ml tubes with silica beads to ensure desiccation and preservation during transport [48]. DNA was extracted using the HigherPurity^™^ Stool DNA Isolation Kit (Canvax Biotech) following the manufacturer’s protocol and stored at −20 °C.

#### Nanopore long-read amplicon sequencing

The *rrn* operon and the full-length 16S rRNA gene were amplified from each DNA sample in separate PCR reactions. Primers 16S_27F (AGRGTTYGATYMTGGCTCAG) and 23S_2490R (CGACATCGAGGTGCCAAAC) were used for the operon [49, 50], whilst 16S_27F (AGRGTTYGATYMTGGCTCAG) and 16S_1391R (GACGGGCGGTGWGTRCA) were used for the full-length 16S gene [50]. Primers included unique barcodes (Table S3) for multiplexing.

The 20 µl PCR reaction for the full-length 16S rRNA gene was performed with 0.4 µl of Platinum SuperFi II (Thermo Fisher Scientific Inc., MA, USA), 4 µl of SuperFi II PCR buffer (Thermo Fisher Scientific Inc., MA, USA), 0.4 µl of dNTPs mix (10 mM), 1 µl of each primer (10 µM), 12.2 µl of nuclease-free water and 1 µl of DNA template. Thermocycling conditions were initial denaturation of 98 °C for 3 min, followed by 30 cycles of 98 °C for 30 s, 60 °C for 30 s and 72 °C for 2 min, with a final extension of 72 °C for 5 min. The 20 µl PCR reaction for the *rrn* operon was performed with 0.4 µl of Platinum SuperFi II (Thermo Fisher Scientific Inc., MA, USA), 4 µl of SuperFi II PCR buffer (Thermo Fisher Scientific Inc., MA, USA), 0.4 µl of dNTPs mix (10 mM), 1.5 µl of each primer (10 µM), 11.2 µl of nuclease-free water and 1 µl of DNA template. The thermocycling conditions were 98 °C for 3 min, followed by 30 cycles of 98 °C for 45 s, 60 °C for 45 s and 72 °C for 3 min, with a final extension of 72 °C for 6 min. A high-fidelity polymerase was used to minimize amplification errors.

PCR amplification was confirmed through electrophoresis on a 1% agarose gel. The resulting amplicons were purified using magnetic beads (1x v/v, Ampure XP, Beckman Coulter). Sample concentration and purity were assessed using a DeNovix DS-11 spectrophotometer (DeNovix Inc, DE, USA) and quantified using a Qubit^™^ dsDNA high sensitivity (Thermo Fisher Scientific Inc., MA, USA). For sequencing, 50–100 fmol of pooled DNA (up to six samples per pool) was used for library preparation with the Ligation Sequencing Kit V14 (SQK-LSK114). Each library was sequenced using Flongle flow cells (R10.4.1) on a MinION Mk1C device until pore exhaustion.

Raw sequencing data were basecalled using Dorado v0.8.1 with the super-high accuracy (‘SUP’) model (sequence details on Table S4). Reads were then filtered using Nanoq v0.10.0 to retain only those with a minimum quality score of Q15 (Table S5). Filtered reads were demultiplexed using seqdemu (https://github.com/hsgweon/seqdemu). Primers were removed with Cutadapt v4.9. Subsequently, reads were filtered by length using SeqKit v2.8.2. The acceptable length range for the full-length 16S rRNA gene (1,300–1,950 bp) was based on the silva database v138.2 [51], whilst the range for the operon (3,500–8,200 bp) was determined based on previous research [38] and the maximum length observed in the op_GROND_refseq database. Samples with fewer than 10,000 processed reads were excluded from further analysis. Sequence details per sample are available in Table S6. Unique sequences were identified by dereplication using VSEARCH v2.30.0 [52], and the resulting cluster file was used to generate an OTU table with the uc2otutable.py script.

#### Illumina short-read amplicon sequencing

The V4 HVR of the 16S rRNA gene was amplified using the Earth Microbiome Project primer pair 515F (GTGYCAGCMGCCGCGGTAA) [53] and 806R (GGACTACNVGGGTWTCTAAT) [54]. The 25 µl PCR reaction consisted of 12.5 µl of JumpStart^™^ Taq ReadyMix^™^ (Sigma-Aldrich), 0.5 µl of each primer (10 µM), 8.5 µl of nuclease-free water and 3 µl of DNA template. Thermocycling conditions were initial denaturation at 94 °C for 3 min, followed by 30 cycles of 94 °C for 45 s, 50 °C for 60 s and 72 °C for 90 s with a final extension at 72 °C for 10 min. PCR products (~300 bp) were visualized on a 1% agarose gel. After the PCR products were purified with magnetic beads, they were pooled in equimolar concentrations and sequenced by Novogene (Cambridge, UK) on the Illumina MiSeq platform using a v2 reagent kit (2×250 bp PE). All 64 samples were sequenced together in a single flow cell to ensure technical consistency. Raw paired-end reads were quality-filtered using TrimGalore (https://github.com/FelixKrueger/TrimGalore) and processed through the DADA2 pipeline v1.14.1 [55] in R. To ensure a standardized and reproducible analysis, all denoising, merging and chimaera removal steps were performed using the pipeline’s default parameters.

#### Construction of custom reference databases for taxonomic assignment

To ensure consistent and accurate taxonomic assignments for the experimental data, three region-specific reference databases (*rrn* operon, full-length 16S and V4) were constructed from the extra curated GROND_refseq database. This process involved a targeted normalization specifically for the three markers sequenced in our experimental workflow, distinct from the global normalization used for the *in silico* benchmarking in the aforementioned Database curation and normalisation section. For this construction, the datasets op_GROND_refseq (76,422 sequences), 16S_GROND_refseq (76,382 sequences) and the V4 HVR (76,262 sequences) were utilized. To maintain consistency across the experimental comparison, any taxon for which the V4 region could not be extracted was removed from all three datasets using the fastaretrieveseqs.py script, resulting in 76,262 matched taxa. By performing this targeted re-filtering, we were able to retain a more comprehensive reference set (76,262 taxa) for the faecal microbiota analysis than would have been possible using the broader 54,522-taxon set required for the multi-HVR *in silico* comparison. Each of the three resulting databases was dereplicated using VSEARCH v2.30.0 [52] to identify unique sequences. This resulted in final databases containing 76,262 representative taxa for the *rrn* operon, 46,265 for the full-length 16S and 13,408 for the V4 region. The databases were then formatted for classifier training using the grond2refdb.py script.

#### Taxonomic assignment

Taxonomic classification of all OTUs and ASVs was performed using OpAssign, a wrapper for the Ribosomal Database Project (RDP) Classifier [56] available on GitHub (https://github.com/hsgweon/opassign). OpAssign employs the naïve Bayesian classification algorithm of the RDP classifier, which uses an 8-nucleotide k-mer approach. The custom-trained databases described in the above Construction of custom reference databases for taxonomic assignment section were used for the respective assignments. The OpAssign workflow requires an OTU/ASV table and a corresponding FASTA file as input. The output provides taxonomic assignments for each sequence with an associated bootstrap confidence score from 100 trials. Only taxonomic assignments with a bootstrap confidence of ≥80% at each taxonomic level were retained for downstream analysis. This filtering was performed using the reformatRDPTaxonomy.py script.

#### Comparative analysis of marker resolution from Nanopore operon data

To compare the taxonomic resolution of the different genetic markers whilst minimizing platform-specific sequencing bias, full-length 16S rRNA and HVR were bioinformatically extracted from the 56 processed *rrn* operon datasets. This internal bias control ensures that the genetic marker is the sole variable by using the exact same parent read set, thereby eliminating confounding platform or primer bias. Of the initial 1,080,148 processed *rrn* operon sequences, 1,059,993 (98.13%) yielded a successfully identified SSU ribosomal region using Infernal v1.1.5 [40] within the acceptable length range (1,300 to 1,950 bp) (Table S7).

Next, HVRs were extracted from these newly isolated full-length 16S sequences by aligning them against the HVR-specific blast databases (described in 2.1.1) using blastn v2.13.0. The retrieved HVR sequences were then length-filtered according to the ranges specified in Table S1, resulting in an average retention of 968,344 sequences (89.65%) across all regions. To ensure coherence, any parent operon sequence from which an HVR could not be successfully retrieved was excluded from all datasets (operon, full-length 16S and all HVRs) for that comparison, using the fastaretrieveseqs.py script. For five of the HVRs (V1V2, V1V3, V2V3, V3V4B and V6V8), some samples yielded fewer than 10,000 extracted sequences and were therefore excluded from the analysis for these specific regions (Table S7). After extraction, sequences were combined by target region, dereplicated using VSEARCH v2.30.0 [52] and used to generate OTU tables for taxonomic assignment and comparison. Final sequence counts before and after clustering are detailed in Table S7.

## Results

### *In silico* analysis: the operon demonstrates superior resolving power and taxonomic accuracy

To establish a theoretical baseline for marker performance, an *in silico* analysis was performed on a curated database. After initial filtering and normalization, a final dataset of 54,522 sequences, each present across the *rrn* operon, the full-length 16S rRNA gene and 11 different HVRs, was used for direct comparison.

#### The *rrn* operon possesses the highest intrinsic resolving power

**Fig. 2.**
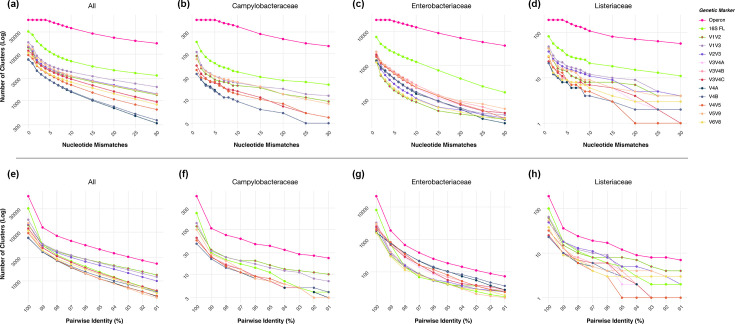
The *rrn* operon consistently resolves into more sequence clusters than the 16S gene or its HVRs. The total number of sequence clusters is plotted against progressively relaxed similarity thresholds for the operon, the full-length 16S gene and 11 HVRs. To improve readability across a wide dynamic range, the Y-axis is log10-scaled with tick labels (e.g. 300, 1,000 and 3,000) reflecting the original untransformed sequence cluster counts. The top row (**a–d**) shows cluster formation as a function of the number of allowed nucleotide mismatches. The bottom row (**e–h**) shows cluster formation as a function of a decreasing PID threshold (%). Analyses are shown for the full dataset (**a, e**) and for subsets of three key pathogenic families: Campylobacteraceae (**b, f**), Enterobacteriaceae (**c, g**) and Listeriaceae (**d, h**). In all eight panels, the 16S-ITS-23S operon (top magenta line) consistently resolves into a substantially higher number of clusters than the full-length 16S gene (green line) or any of the HVRs, confirming its superior ability to differentiate between closely related sequences.

The intrinsic resolving power of each marker was first evaluated by measuring its ability to differentiate sequences into distinct clusters. Across all tested thresholds, the *rrn* operon consistently produced the highest number of clusters (Fig. 2). When allowing for up to 30 nucleotide mismatches, the operon dataset resolved into 17,078 clusters. This represents a 3.92-fold greater resolution than the full-length 16S rRNA gene (3,469 clusters) and a 7.70- to 51.55-fold greater resolution than the HVRs, which ranged from V1V3 (1,963 clusters) to V4A (325 clusters). This trend was even more pronounced within specific pathogenic families. For Enterobacteriaceae, the operon’s resolving power was over 22-fold higher than the 16S gene at 30 mismatches. Similarly, for Listeriaceae and Campylobacteraceae, the operon maintained 4.18-fold and 6-fold more clusters, respectively, than the full-length 16S gene under the same conditions. Whilst we acknowledge that a fixed mismatch threshold represents a varying percentage of the total length across different markers, this initial analysis highlights the absolute sequence divergence captured by the operon. Critically, this superior performance was maintained when clustering by PID. At a relaxed 91% PID threshold, the operon maintained 2,266 clusters. This represents a 2.72-fold higher resolution compared to the full-length 16S rRNA gene (609 clusters). Whilst some HVRs showed improved performance at this specific threshold (e.g. V1V2 with 1,328 clusters), the operon still maintained the highest number of clusters overall, with up to a 3.99-fold advantage over the lowest-performing HVR (V5V9 with 454 clusters). The consistency of the operon’s top performance across both mismatch- and PID-based metrics reinforces the conclusion that it captures a greater degree of true sequence diversity. Whilst a ~4.5 kb sequence mathematically allows for a higher number of unique variants compared to shorter regions, our analysis demonstrates that this additional sequence provides a substantial biological advantage for species differentiation. Crucially, because PID is a length-normalized metric, these results demonstrate that the operon’s superior resolving power is driven by the greater amount of genetic information within the full *rrn* operon. This consistent advantage in sequence differentiation remains robust even when normalized for sequence length, as demonstrated by the PID thresholds.

#### The operon’s high resolution translates to superior taxonomic accuracy

**Fig. 3.**
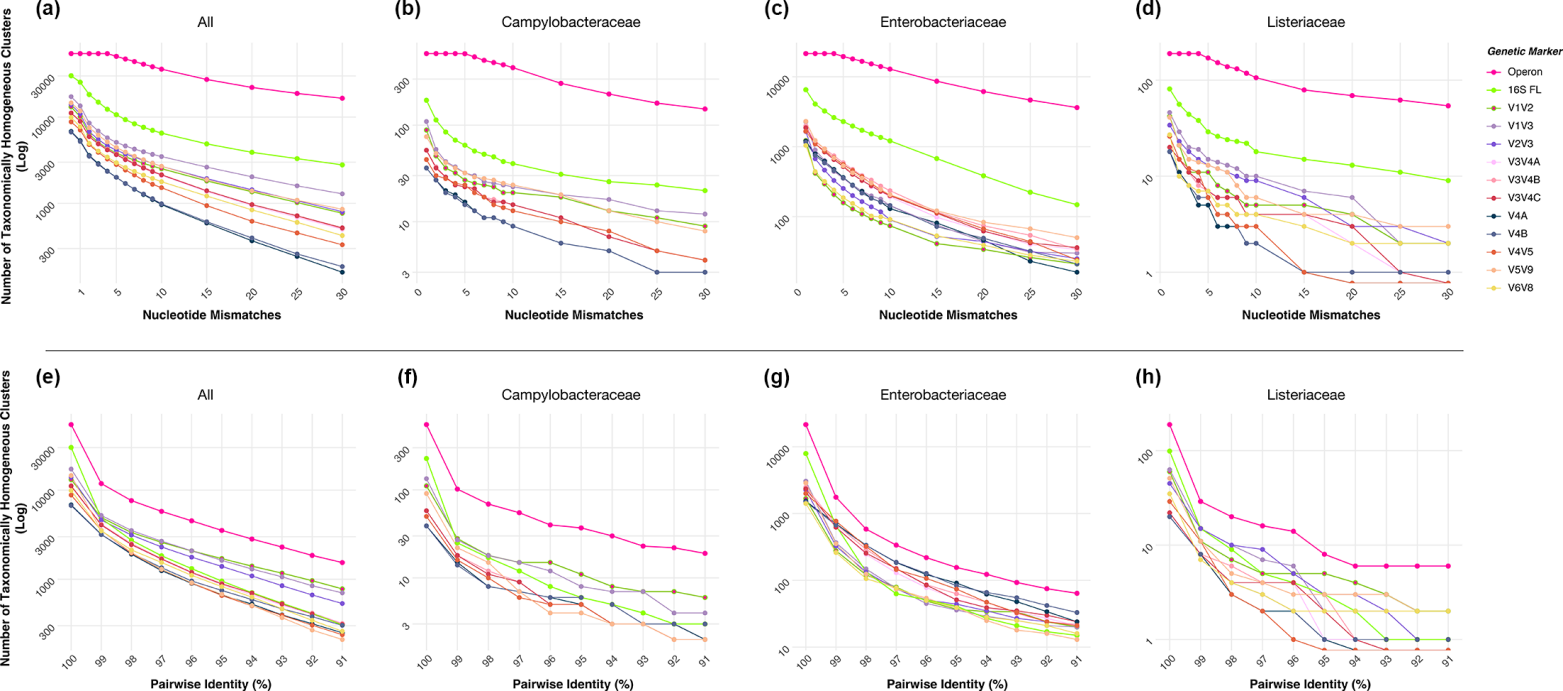
Taxonomic accuracy of genetic markers measured by the formation of monospecies clusters. The analysis assesses each marker’s ability to form clusters that are accurate at the species level. The number of taxonomically monospecies clusters – defined as clusters containing sequences that all originate from a single species – is plotted against progressively relaxed similarity thresholds. To improve readability across a wide dynamic range, the Y-axis is log10-scaled with tick labels (e.g. 300, 1,000 and 3,000) reflecting the original untransformed sequence cluster counts. The top row (**a–d**) shows the number of monospecies clusters as a function of allowed nucleotide mismatches. The bottom row (**e–h**) shows the number as a function of a decreasing PID threshold (%). Analyses are shown for the full dataset (**a, e**) and for subsets of three key pathogenic families: Campylobacteraceae (**b, f**), Enterobacteriaceae (**c, g**) and Listeriaceae (**d, h**). The operon consistently maintains the highest number of monospecies clusters, showing a unique resistance to collapsing into mixed-species clusters, especially at relaxed thresholds. This highlights its superior reliability for accurate species-level classification.

High resolving power is only beneficial if the clusters formed are taxonomically meaningful. Therefore, the accuracy of each marker was assessed by quantifying the number of monospecies clusters – defined as clusters where all constituent sequences belong to a single species (Fig. 3).

The *rrn* operon maintained 100% of its clusters as monospecies with up to four allowed mismatches, which was not achieved by any other marker. Even at the highly relaxed threshold of 30 mismatches, 96.8% (16,529 of 17,078) of operon clusters remained monospecies. In contrast, the full-length 16S gene maintained only 80.5% monospecies clusters, whilst the HVRs performed much worse, dropping to between 48.9% (V4A) and 65.7% (V1V3). This shows that the HVRs and, to a lesser extent, the full-length 16S gene are far more prone to incorrectly grouping sequences from different species as similarity thresholds are relaxed. This superior accuracy was consistent across the pathogenic families. For Enterobacteriaceae, the operon maintained 95.9% monospecies clusters at 30 mismatches, compared to 91.9% for the 16S gene. For Listeriaceae, several HVRs failed to produce any monospecies clusters at 30 mismatches, whilst the operon still maintained 94.7% accuracy.

The PID clustering reinforced the improved resolution of the *rrn* operon. Even with the lower threshold of 91% PID, *rrn* operon clustering yielded 67.8% of monospecies clusters (1537 of 2,266), whereas only 50.2% of the full-length 16S clusters remained as monospecies, and HVRs ranged from 46.3% to 58%. This pattern was consistently observed in the tested pathogenic families. Clustering at 91% PID, the Enterobacteriaceae family still presented 87.7% (64 of 73) monospecies clusters with the *rrn* operon, compared to 88.2% (15 of 17 clusters) for the full-length 16S. This apparent similarity is caused by the smaller number of 16S clusters, with the *rrn* operon still retaining around fourfold more monospecies clusters than the 16S. At the same PID threshold, the Listeriaceae and Campylobacteraceae families had 85.7 and 82.6% of monospecies clusters, respectively, with the *rrn* operon, compared to 50 and 75% for the full-length 16S. These results provide strong theoretical evidence that the *rrn* operon is not only more powerful at differentiating sequences but is also significantly more reliable for accurate species-level classification.

### Experimental validation in rat faecal samples

To validate the *in silico* findings, the performance of the three marker strategies (*rrn* operon, full-length 16S and 16S V4) was tested on 64 rat faecal samples using the relevant sequencing technologies.

#### Summary of sequencing output

Nanopore sequencing on 15 Flongle flow cells generated a total of 4,710,320 raw reads for the *rrn* operon, with a mean quality score of Q16.65. For the full-length 16S rRNA gene, 14 Flongle flow cells produced 3,119,656 raw reads with a mean quality score of Q17.42. After quality filtering (Q15), the mean quality scores improved to Q19.01 and Q19.07, respectively. Following all processing steps, 56 samples yielded 1,060,005 high-quality operon OTUs (all unique), and 59 samples yielded 1,234,582 high-quality full-length 16S OTUs. For the Illumina sequencing of the 16S V4 region, all 64 samples were successfully processed, yielding a total of 1,347,568 paired-end sequences that clustered into 8,412 unique ASVs.

#### The operon delivers the highest rate of species-level assignment in complex samples

**Fig. 4.**
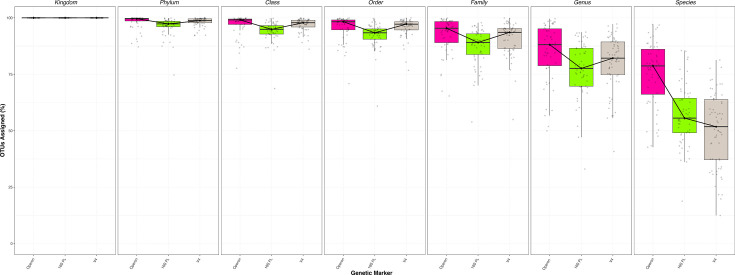
Comparison of taxonomic assignment rates across three different experimental sequencing methods. Experimental performance of three distinct sequencing strategies for taxonomic classification of rat faecal microbiota. The percentage of total OTUs or ASVs assigned a taxonomy is presented for each taxonomic rank. Boxplot elements are defined as follows: the centre line represents the median, the box bounds the interquartile range (IQR; 25th to 75th percentiles), and the whiskers extend to the largest and smallest values within 1.5×IQR of the hinge. Individual data points are independent samples. The solid black lines connect medians across taxonomic ranks to highlight the overall trend in classification drop-off. The three methods compared are (1) 16S-ITS-23S operon long-read sequencing using Nanopore R10.4.1 (magenta), (2) full-length 16S rRNA long-read sequencing using Nanopore R10.4.1 (green) and (3) 16S V4 HVR short-read sequencing using Illumina (grey).

To validate the *in silico* findings in an experimental context, the taxonomic assignment performance of the three sequencing strategies was directly compared using the rat faecal samples. The results show that the *rrn* operon method provides a superior level of taxonomic resolution across all ranks, with the performance gap becoming most pronounced at the species level (Fig. 4). Whilst all three methods successfully assigned nearly all sequences at the kingdom and phylum ranks, their ability to resolve deeper taxonomies diverged significantly. The 16S V4 HVR marker, sequenced on the Illumina platform, showed a dramatic decline in performance, assigning a species-level taxonomy to a mean of only 18.52% of its unique ASVs. The full-length 16S rRNA gene, sequenced with Nanopore technology, performed substantially better, assigning 62.10% of its OTUs to a species. The *rrn* operon method, however, demonstrated the highest performance, successfully assigning a species-level identity to 75.20% of its OTUs. This result provides strong experimental evidence that corroborates the *in silico* analyses, confirming that the additional genetic information in the full operon translates directly to a more powerful and accurate classification tool for high-resolution microbial surveillance.

#### Operon superiority is independent of sequencing platform bias

**Fig. 5.**
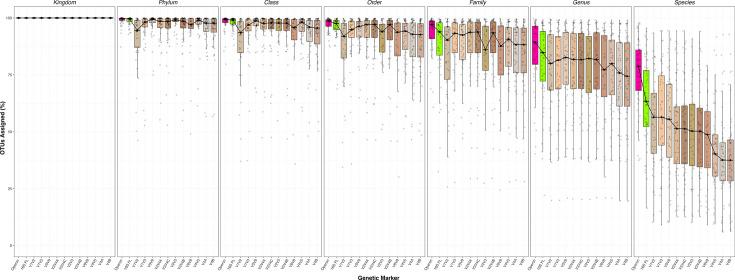
Comparison of taxonomic assignment rates for different genetic markers extracted from a single sequencing dataset. Taxonomic resolution was evaluated whilst controlling for sequencing platform bias. The percentage of total OTUs assigned a taxonomy is shown for each taxonomic rank. The comparison features different genetic markers (x-axis), including the full 16S-ITS-23S operon (magenta), the full-length 16S rRNA gene (green) and various HVRs (different shades of brown). All markers were bioinformatically extracted from the same parent operon reads generated by Nanopore sequencing, ensuring a direct comparison of their intrinsic informational content. Boxplot elements are defined as follows: the centre line represents the median, the box bounds the IQR (25th to 75th percentiles), and the whiskers extend to the largest and smallest values within 1.5×IQR of the hinge. Individual data points represent independent samples. The solid black lines connect the medians across the different genetic markers to illustrate the overall trend in taxonomic resolution at each rank.

To definitively attribute the observed performance differences to the genetic information within the markers themselves, rather than to biases from different sequencing technologies, primer choice and library preparation, a comparative analysis was performed using markers bioinformatically extracted from the same high-quality Nanopore operon reads. This internal bias control was designed to decouple information content from the confounding variables of platform-specific error profiles and primer bias. Whilst we acknowledge that this approach removes the inherent per-base accuracy advantages associated with short-read platforms, it provides a necessary level playing field to evaluate the intrinsic resolving power of each genetic region as the sole variable. The results confirm that the *rrn* operon provides the most comprehensive taxonomic classification at all ranks, demonstrating that its superior performance is an intrinsic property driven by its greater genetic information content (Fig. 5).

As expected, the proportion of assigned OTUs decreased for all markers as the taxonomic rank became more specific, from near-complete assignment at the phylum level to much lower rates at the species level. Critically, the performance gap between the markers widened significantly at these deeper taxonomic ranks. At the species level, the *rrn* operon achieved the highest median assignment rate. The full-length 16S rRNA gene performed second best but was substantially lower than the operon. In contrast, all HVRs performed significantly worse, exhibiting both lower median assignment rates and greater variability across samples and between different HVR regions. This finding demonstrates that the superior performance of the *rrn* operon is an intrinsic property driven by its greater genetic information content.

## Discussion

This study provides a comprehensive evaluation of the full *rrn* operon as a genetic marker for species-level bacterial classification. By systematically combining *in silico* modelling with direct experimental application to rat faecal microbiota, we demonstrate that the operon offers a significant improvement in taxonomic resolution compared to both the full-length 16S rRNA gene and the standard V4 16S HVR marker. These findings provide a validated framework for leveraging high-accuracy long-read sequencing as a tool for high-resolution surveillance.

### Resolving power vs. taxonomic accuracy

The *in silico* analysis systematically assessed two key properties of a genetic marker: its resolving power and its taxonomic accuracy. The *rrn* operon captures significantly more sequence diversity than the 16S rRNA gene or its subregions, confirming its superior resolving power (Fig. 2). Whilst this study serves as a foundational benchmarking of taxonomic resolution rather than a direct diagnostic validation for specific pathogen-sister pairs, such high resolution is a prerequisite for distinguishing between closely related species where the 16S gene alone often fails, particularly within medically important genera [4, 11, 57, 58]. This high resolution is largely attributable to the increased phylogenetic information contained within the full ~4.5 kb region [59, 60], offering potential for subspecies-level differentiation [33]. Critically, the operon’s superior resolving power remains robust when evaluated via PID. Although a fixed count of nucleotide mismatches represents a larger percentage of a short HVR than of a 4.5 kb operon, PID provides a length-normalized comparison that directly reflects sequencing error profiles. The maintenance of superior resolution under these normalized conditions definitively demonstrates that the operon’s performance is driven by its greater genetic information content rather than a mathematical artefact of its length.

However, high resolution is only valuable if it is taxonomically accurate. Our analysis of monospecies (single-species) clusters revealed the operon’s superiority in this regard (Fig. 3). It maintained a high proportion of monospecies clusters even at relaxed similarity thresholds, indicating that the genetic distance between species consistently exceeds intra-species variation – a crucial trait for a reliable marker. This addresses the limitations of other markers: whilst 16S HVRs often fail by over-clustering (i.e. lumping distinct species), the full-length 16S can also fail by co-clustering (i.e. incorrectly grouping species that share >99% 16S identity) [11, 57]. The *rrn* operon's greater information content provides the necessary framework to overcome these barriers, offering a more stable basis for high-resolution surveillance. Furthermore, our study aligns with Johnson *et al*. [11] by demonstrating that when clustering is performed to account for intragenomic variants, the operon provides a far more robust and accurate basis for forming these species-level groups than the 16S gene alone.

Whilst this *in silico* validation is constrained by the accuracy of public reference databases, which can be inconsistent [59–63], it provides a robust theoretical framework for the *rrn* operon’s potential. In this context, adopting alternative taxonomic frameworks such as the Genome Taxonomy Database (GTDB) [64] – which prioritizes genome-based phylogeny over legacy nomenclature – may resolve existing discrepancies where genomic siblings (e.g. *Escherichia coli* and *Shigella* [65]) are currently classified as distinct species under the NCBI RefSeq system. Such a transition would likely further improve the monospecific clustering metrics observed for high-resolution markers like the *rrn* operon. Furthermore, as genomic databases such as GROND continue to be updated and standardized [37], the accuracy of long-read taxonomic profiling will continue to improve. Ensuring that reference databases are regularly updated to reflect the most current and phylogenetically consistent genomic data remains a prerequisite for the reliable implementation of *rrn* operon sequencing in clinical and environmental surveillance. Our experimental results from rat faecal samples further support this by demonstrating the practical feasibility of retrieving and classifying full-length operon sequences from complex communities, providing a proof-of-principle for its future application in high-resolution profiling.

Despite this superior resolving power, it is important to consider the biological limits of using a ribosomal marker to differentiate pathogenic bacteria from their non-pathogenic relatives. For many genera, such as *Listeria* or many *Campylobacter*, the *rrn* operon provides clear species-level separation that effectively distinguishes primary pathogens from benign commensals. However, in cases where pathogenicity is determined by mobile genetic elements, such as the Shiga toxin-encoding prophages in *E. coli* or the pXO1 and pXO2 plasmids in *Bacillus anthracis*, ribosomal markers alone remain insufficient for full pathotype characterization [66, 67]. In these instances, the *rrn* operon serves as a highly accurate taxonomic scaffold that can be augmented by targeted sequencing of specific virulence factors. Thus, whilst the *rrn* operon represents a significant advancement for high-resolution surveillance, it should be viewed as a powerful tool for species-level screening that may, in specific clinical contexts, require complementary genomic data for definitive pathotyping.

### Experimental validation in complex host-associated microbiota

To determine if the operon’s theoretical advantages translate to a realistic application, we sequenced rat faecal samples. The initial analysis showed that the operon enabled species-level classification for 75% of unique sequences, a marked improvement over full-length 16S (62%) and the V4 region (18.5%) (Fig. 4). However, direct comparison of these methods is complicated by potential biases from different primers and sequencing platforms, i.e. Illumina vs. Nanopore [68–73].

To isolate the true effect of the genetic marker itself, we performed an internal control by computationally extracting 16S and HVR sequences from the same high-quality operon reads (Fig. 5). This bias-controlled analysis confirmed the experimental findings: the full operon consistently yielded the highest rate of taxonomic assignment at every rank. This demonstrates that its superior performance is a genuine function of its greater genetic information, not an experimental artefact. Our results align with the broader literature, confirming the poor species-level resolution of the V4 region [11, 74, 75] and the substantial improvement offered by full-length 16S sequencing [20, 74, 76]. However, this study’s key contribution is demonstrating that sequencing the entire operon provides a further, significant leap in resolution. This gain is critical for surveillance, offering the precision needed to distinguish closely related pathogens. Furthermore, this targeted amplicon approach is highly efficient for host-associated studies, avoiding the costly issue of host DNA contamination that limits shotgun metagenomic methods [10].

### Technological advancements as an enabling factor for high-fidelity long-read amplicons

The success of this approach is fundamentally predicated on recent and rapid advancements in Oxford Nanopore sequencing technology. Early iterations of this technology were characterized by high error rates (5–15%), which rendered them unsuitable for amplicon sequencing, where single-nucleotide differences can be critical for taxonomic differentiation [16, 21–25]. This limitation is why the field has long relied on the high per-base accuracy of Illumina sequencing. However, the introduction of the R10.4.1 flow cell chemistry, combined with advanced basecalling models such as Dorado ‘SUP’, has pushed the modal accuracy of single-pass reads to >99% [17, 19, 77]. Whilst a detailed benchmarking of the technical biases between sequencing platforms was beyond the scope of this research, such investigations remain a vital area for future work to complement our findings on marker-specific resolving power.

This study serves as a practical demonstration that this technological maturation has crossed a critical threshold. The accuracy is now sufficient to resolve the subtle differences between closely related bacterial species using long amplicons, effectively combining the length advantage of third-generation sequencing with the accuracy approaching that of second-generation platforms. Whilst others have validated this accuracy in the context of whole-genome sequencing [18, 78], this study validates its application for high-resolution, targeted surveillance.

Whilst this study validates the accuracy of long-read *rrn* sequencing, there remain practical trade-offs regarding laboratory implementation. Standard short-read platforms currently offer higher per-run throughput and established economies of scale, which may be more cost-effective for large-scale, centralized batching of samples. In contrast, long-read technologies such as the MinION provide a significantly reduced laboratory footprint and on-demand real-time data generation [79, 80]. By utilizing low-output, entry-level consumables such as the Flongle flow cell used in this study, researchers can achieve high-resolution profiling with minimal upfront sample requirements. This offers a compelling advantage for rapid-response scenarios where time to result is prioritized over total sample throughput. Furthermore, the development of custom bioinformatics tools like this study’s OpAssign is a necessary step to handle the analytical challenges of variable-length, error-profile-specific long reads, as established pipelines for short reads are not directly applicable.

Despite these advancements, several bottlenecks currently prevent *rrn* operon sequencing from becoming the standard for microbiota profiling. Historically, the primary hurdles were high error rates and a lack of curated, full-length operon databases – a gap only recently addressed by resources like GROND [37]. However, laboratory challenges remain significant, particularly the difficulty of consistently amplifying large ~4.5 kb fragments from degraded DNA or low-biomass environmental samples. Furthermore, the bioinformatics community is still in the process of standardizing workflows to account for the unique intragenomic variation of the full operon. As these laboratory and computational barriers are addressed, we anticipate that the *rrn* operon will increasingly supersede shorter markers for high-resolution bacterial characterization.

### Considerations, limitations and future directions

Whilst this study demonstrates the clear advantages of operon sequencing, its primary limitations define the next steps for the field. The accuracy of any classification method is constrained by its reference database; therefore, the continued expansion of high-quality, full-operon databases beyond the current NCBI RefSeq bias is critical for classifying novel or uncultivated taxa. In this context, another taxonomic framework strategy such as the GTDB, which uses genome-based taxonomy for classification, might be beneficial [64]. A significant consideration for high-resolution profiling is the presence of multiple, non-identical *rrn* operons within a single bacterial genome. For example, organisms such as *E. coli* possess up to seven operon copies with subtle sequence variations [81]. This intragenomic heterogeneity can lead to false splits where different operons from the same strain are assigned to distinct sequence clusters. However, as demonstrated in our *in silico* analysis, the *rrn* operon’s information content ensures that these clusters predominantly remain monospecies. This indicates that the genetic distance between species typically exceeds the variation found within a single genome. Whilst other markers like 16S HVRs may ‘hide’ this variation by lumping different species into a single cluster, the *rrn* operon provides the resolution necessary to identify these variants whilst maintaining accurate species-level boundaries. As curated operon databases grow to include known within-genome variants [37, 82], they will further reduce ambiguous alignments caused by documented intragenomic variation.

Looking forward, future validation efforts should focus on targeted benchmarking of high-consequence pathogens against their closely related non-pathogenic relatives. Further, the implications of this work are significant, offering an immediate framework to enhance bacterial surveillance in diverse ‘One Health’ contexts, from wastewater and food safety to livestock and wildlife reservoirs. Transitioning this powerful method into a standard tool will require community-wide efforts to standardize protocols and develop automated, user-friendly bioinformatics pipelines, making it fully accessible to public health laboratories and researchers worldwide.

## Conclusion

In conclusion, this study demonstrates that sequencing the full ~4.5 kb *rrn* operon provides a significant advancement in taxonomic resolution over the 16S rRNA gene and its HVRs. Our *in silico* and experimental data confirm that the operon captures the genetic diversity required to achieve species-level differentiation within complex microbiota, particularly in medically relevant families such as Enterobacteriaceae and Listeriaceae. Whilst this research serves as a theoretical and practical benchmarking of resolving power rather than a direct diagnostic validation, it establishes the *rrn* operon as a robust framework for high-resolution bacterial profiling. By providing a more accurate taxonomic scaffold, *rrn* operon sequencing offers a powerful tool for surveillance and the differentiation of closely related species, although complementary genomic data may still be required for the characterization of specific pathotypes.

## Supplementary material

10.1099/mgen.0.001793 Supplementary Material 1.

## Abbreviations

HVR: hypervariable region
IQR: interquartile range
ITS: internal transcribed spacer
OTU: operational taxonomic unit
PID: pairwise identity
RDP: Ribosomal Database Project
